# Effect of magnetron sputtering parameters and stress state of W film precursors on WSe_2_ layer texture by rapid selenization

**DOI:** 10.1038/srep36451

**Published:** 2016-11-04

**Authors:** Hongchao Li, Di Gao, Senlin Xie, Jianpeng Zou

**Affiliations:** 1 State Key Laboratory of Powder Metallurgy, Central South University, Changsha 410083, China; 2 Chongyi Zhangyuan Tungsten Industry Corporation Limited, Ganzhou 341300, China

## Abstract

Tungsten diselenide (WSe_2_) film was obtained by rapid selenization of magnetron sputtered tungsten (W) film. To prevent WSe_2_ film peeling off from the substrate during selenization, the W film was designed with a double-layer structure. The first layer was deposited at a high sputtering-gas pressure to form a loose structure, which can act as a buffer layer to release stresses caused by WSe_2_ growth. The second layer was deposited naturally on the first layer to react with selenium vapour in the next step. The effect of the W film deposition parameters(such as sputtering time, sputtering-gas pressure and substrate bias voltage)on the texture and surface morphology of the WSe_2_ film was studied. Shortening the sputtering time, increasing the sputtering-gas pressure or decreasing the substrate bias voltage can help synthesize WSe_2_ films with more platelets embedded vertically in the matrix. The stress state of the W film influences the WSe_2_ film texture. Based on the stress state of the W film, a model for growth of the WSe_2_ films with different textures was proposed. The insertion direction of the van der Waals gap is a key factor for the anisotropic formation of WSe_2_ film.

Tungsten diselenide (WSe_2_) belongs to a class of transition-metal dichalcogenide materials with a graphite-like layered microstructure, in which atoms that form each layer are bonded covalently, whereas adjacent layers are bound weakly by van der Waals forces^1^,^2^. WSe_2_ film has attracted intensive research interest because of its excellent applications, such as in photoelectrochemistry^3^, photovoltaic solar cells^4^, dry lubricants^5^, catalysts^6^, and the hydrogen-evolution reaction^7^.

In most applications, synthesis of high-textured WSe_2_ films is essential. Two types of WSe_2_ films have been reported: C-axis parallel to the substrate (C-axis // substrate) and C-axis perpendicular to the substrate (C-axis ⊥ substrate)^8^,^9^. The C-axis // substrate-textured WSe_2_ film is useful in catalysts and in the hydrogen-evolution reaction, and the C-axis ⊥ substrate-textured WSe_2_ film is necessary for use in photoelectrochemistry and photovoltaic solar cells^3^,^4^.

Soft selenization^10^, physical vapor transport^11^, electrodeposition^12^, pulsed laser deposition^13^, selenium–oxygen ion exchange^14^, and atmospheric pressure chemical vapor deposition^15^ have been reported to fabricate WSe_2_ film. Among these synthetic methods, an environmentally friendly method by soft selenization of the W film has gained superiority and may be ready for potential development.

The biggest problem for WSe_2_ film preparation by soft selenization is the peel off problem. Residual stresses exists in magnetron sputtered W films. These stresses are influenced by the sputtering time, sputtering-gas pressure and substrate bias voltage^16^,^17^. The W film expands during soft selenization due to the different densities (ρ_W_ = 19.3 g/cm^3^, ρ_WSe2_ = 4.8 g/cm^3^). According to Jäger’s report, the WSe_2_ film produced in the reaction is ~3.84 times thicker than the original W film^10^. The expansion will be restricted by the substrate, so the expansion stress can be produced. Once the expansion stress of the WSe_2_ film exceeds a certain value, it will cause the WSe_2_ film to peel completely from the substrate.

A novel idea has been presented to solve this peel off problem. W film precursor is designed with a double-layer structure: the first layer is the buffer layer to relax the residual stress and the second layer is the working layer to react with selenium vapour in the next step. Different W film sputtering deposition parameters (sputtering time, sputtering-gas pressure and substrate bias voltage) were designed to investigate their effect on WSe_2_ film growth. We evaluate the relationship between the W film precursors and the WSe_2_ texture. The residual stress of W film was tested and a model based on the stress state was constructed to determine the influence of W film stress state on WSe_2_ film texture.

## Results

### Effect of W film’s sputtering time on WSe_2_ film

**Figure 1:**
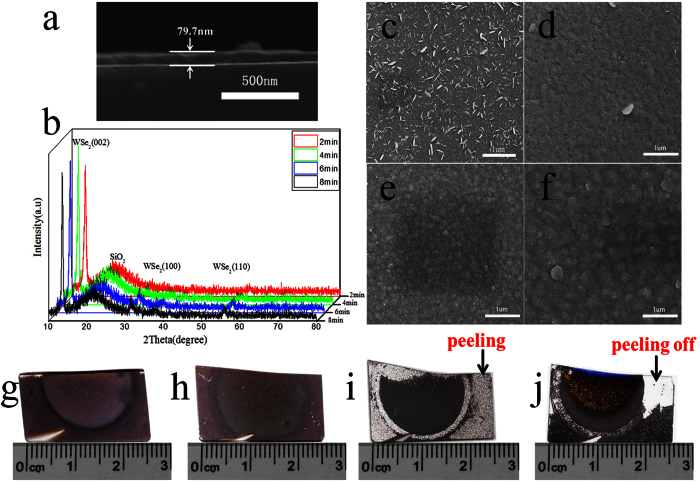
Characterization of WSe_2_ film prepared by selenization of W films with different sputtering time. (**a**) SEM image of the W film’s cross-section, whose sputtering time is 8 min. (**b**) XRD patterns of WSe_2_ films prepared by selenization of W films with different sputtering time. SEM images of WSe_2_ films prepared by selenization of W films with different sputtering time (**c**) 2, (**d**) 4, (**e**) 6 and (**f**) 8 min. Digital photographs of WSe_2_ films prepared by selenization of W films with different sputtering time (**g**) 2, (**h**) 4, (**i**) 6 and (**j**) 8 min.

As reported, the residual stress of the magnetron-sputtering W film is influenced by its thickness. The W film, which is deposited at a low pressure, presents a remarkable evolution of residual stress with increasing film thickness from 25 to 200 nm. Tensile stress appears in the 25 nm-thick W film, whereafter the tensile stress decreases with the increase of W film thickness. When the thickness reaches 50 nm, the stress state changes to a compressive stress and it remains constant for thicknesses up to 200 nm^16^. The same conclusion was obtained by Shen^17^. Based on previous research findings, W film was deposited at a low pressure of 0.2 Pa with different sputtering time. The deposition parameters for the W films are summarized in Table 1.The deposition rate is the film thickness divided by the deposition time. Under the same sputtering conditions, the deposition rate can be regarded as constant and the film thickness is linearly related with sputtering time^18^. The cross-section and a rough thickness of the W film were observed by SEM. Because the thicker the W film is, the smaller the measurement error is. The W film, whose sputtering time is 8 min, is the thickest of the four samples. Therefor it is selected to estimate deposition rate. Figure 1a shows the film thickness is 79.7 nm when the deposition time is 8 min. So the deposition rate is 79.7 nm/8 min≈10 nm/min. The film thickness is about 20, 40, 60 nm when the deposition time is 2, 4, 6 min.

The XRD patterns in Fig. 1b indicate that WSe_2_ films with a 2H-WSe_2_ hexagonal microstructure were synthesized. Peaks were identified according to the standard WSe_2_ PDF card (No. 38–1388). Peaks at 13.62°, 31.41°, and 55.90° correspond to the (002), (100) and (110) crystal planes of WSe_2_, respectively. Because the intensities of the (100) and (110) peaks are very weak compared with that of the (002) peak (Fig. 1b), WSe_2_ films show an obvious (002) preferential orientation. The peak at 20° is the diffraction peak of the SiO_2_ substrate.

The surface morphology of the WSe_2_ films is shown in Fig. 1c–f. When the W film’s sputtering time is 2 min, there are many small platelets on the surface of the WSe_2_ and most of the platelets are embedded vertically in the matrix (Fig. 1c). As the sputtering time increased from 2 to 4 min, the number of platelets embedded in the matrix decreased significantly (Fig. 1d). With increasing sputtering time from 6 to 8 min, no significant change happened and no platelets were embedded vertically in the matrix (Fig. 1e,f). But when the sputtering time is 8 min, some platelets grow in directions nearly parallel to the surface(Fig. 1f).

Digital photographs of the WSe_2_ film are shown in Fig. 1g–j. When the W film’s sputtering time is 2 and 4 min, more perfect WSe_2_ films without apparent pores and cracks can be prepared (Fig. 1g,h). When the W film’s sputtering time is 6 min, there are many pores on the surface of WSe_2_ films and the WSe_2_ film peels slightly off from the substrates (Fig. 1i). When the W film’s sputtering time is 8 min, the peeling off problem is more serious than that for 6-min and part of WSe_2_ film peeled completely off from the substrates (Fig. 1j). The 80-nm-thick W film, whose sputtering time is 8 min, exhibits the maximum compressive stress among the four samples. When the W film reacts with Se vapor to form WSe_2_, the expansion causes the WSe_2_ film to have a larger compressive stress compared with the original W film. The stresses caused by expansion reach a certain value, so the WSe_2_ film peels off from the substrate.

### Effect of W film’s sputtering-gas pressure on WSe_2_ film

To solve the peel off problem of the WSe_2_ film during the selenization reaction, W film with a double-layer structure was designed. The first layer was deposited at a high gas pressure to achieve a loose structure. The loose layer can serve as a buffer layer to release the expansion stress that caused by WSe_2_ growth. The second layer was deposited naturally on the first layer to react with selenium in the next step.

The residual stress of the magnetron sputtered W films is also influenced by the sputtering-gas pressure^16^,^17^. The magnetron sputtered W film undergoes a transition from compression to tension with increasing sputtering-gas pressure. In general, the stress state of the W film is a compressive stress at low pressure. With increasing sputtering-gas pressure, the stress state of the W film changes to a tensile stress. Based on previous research findings, W film was deposited at different sputtering-gas pressures (0.2~2.0 Pa). The thickness of the first buffer layer that deposited at 2.0 Pa for 10 mins is ~210 nm. But the deposition rate is not the same at different sputtering-gas pressure. To exclude the possible influence on the stress from the thickness change, the second layer was deposited at different time to obtain the same ~250 nm-thick film. The total thickness of the W films is ~460 nm.The deposition parameters for the W films are summarized in Table 2.

**Figure 2:**
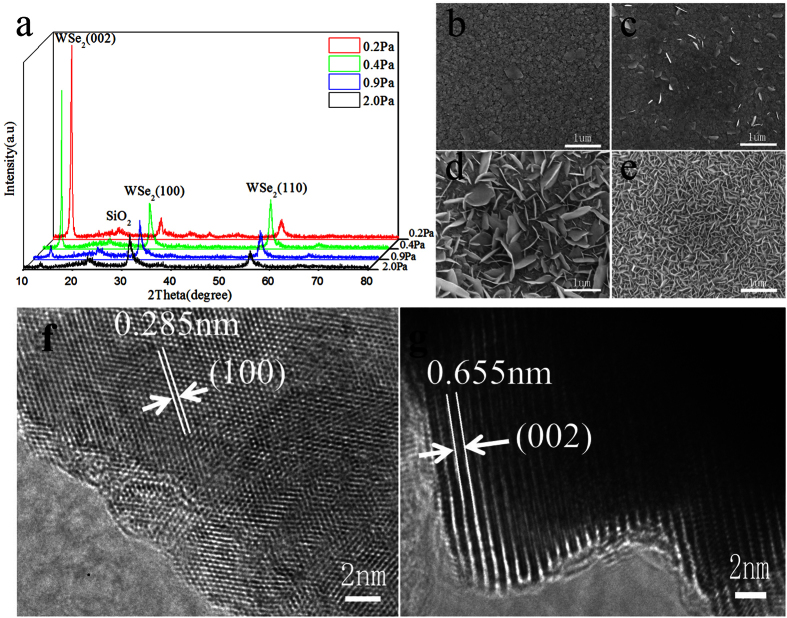
Characterization of WSe_2_ film prepared by selenization of W films deposited at different sputtering-gas pressures. (**a**) XRD patterns of WSe_2_ films prepared by selenization of W films deposited at different sputtering-gas pressures. SEM images of WSe_2_ films prepared by selenization of W films deposited at different sputtering-gas pressures (**b**) 0.2, (**c**) 0.4, (**d**) 0.9, and (**e**) 2.0 Pa. HRTEM of WSe_2_ films prepared by selenization of W films deposited at P_Ar_ for (**f**) 0.2 and (**g**) 2.0 Pa.

The XRD patterns of WSe_2_ films prepared by selenization of W films are shown in Fig. 2a. When the sputtering-gas pressure is 0.2 Pa, the intensities of the (100) and (110) peaks are very weak compared with that of the (002) peak. WSe_2_ film with (002) preferential orientation can be prepared. As the sputtering-gas pressure increases to 0.4 Pa, the intensity of the (002) peak decreases slightly and that of the (100) and (110) peaks increases compared with the sample at 0.2 Pa. As the sputtering-gas pressure increases from 0.4 to 0.9 Pa, the (002) diffraction peaks become weaker. When the sputtering-gas pressure is 2.0 Pa, the (002) peak of the WSe_2_ film almost disappears.

The surface morphology of the WSe_2_ films is shown in Fig. 2b–e. The photographs show striking differences. When the sputtering-gas pressure is 0.2 Pa, WSe_2_ shows a relatively flat surface. Some platelets grow in directions nearly parallel to the surface (Fig. 2b). As the sputtering pressure increases from 0.2 to 2.0 Pa, there are more and more platelets that embedded in the matrix and the number of platelets increased visibly (Fig. 2b–e). When the sputtering-gas pressure is 2.0 Pa, it shows a closely-packed lamellar surface (Fig. 2e).

When the sputtering-gas pressure is 0.2 Pa, the HRTEM micrographs show a hexagonal arrangement of atoms and a somewhat disordered region at the edge of the crystal on the surface of the WSe_2_ film (Fig. 2f). A lattice spacing of 0.285 nm on the surface is attributable to the (100) planes of 2H-WSe_2_. So the C-axis of the WSe_2_ crystal is perpendicular to the substrate.

When the sputtering-gas pressure is 2.0 Pa, it appears that the surface is covered with vertically aligned layers (Fig. 2g). The interlayer spacing of the dark fringes is 0.655 nm, which corresponds to the (002) plane of the hexagonal WSe_2_. So the C-axis of the WSe_2_ crystal is parallel to the substrate.

Further structural characterization provided additional insight into these films. TEM images show that WSe_2_ films contain different textures when the sputtering-gas pressure is different.

### Effect of W film’s substrate bias voltage on WSe_2_ film

The substrate bias voltage can increase the compressive stress or decrease the tensile stress^19^,^20^,^21^. When the substrate is applied with a bias voltage, an electric field is formed between the substrate and the anode. So the argon ions in the plasma are accelerated to reach the substrate. The argon ions’ bombardment will provide sufficient energy for W atom deposition on the W film surface. The W atoms on the surface move inside, which yields a volumetric expansion. This process causes an increase in compressive stress compared with the W film without substrate bias voltage.

To determine whether the number of platelets embedded in the matrix are influenced by the substrate bias voltage, a gas pressure of 0.9 Pa was selected and a substrate bias voltage was used only for the second layer of the W film. The deposition parameters for the W films are summarized in Table 3.

**Figure 3:**
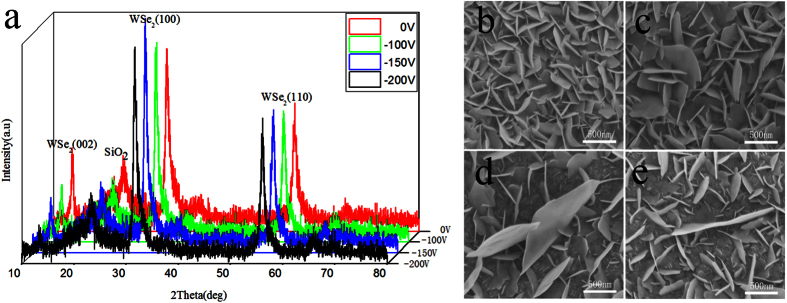
Characterization of WSe_2_ film prepared by selenization of W films with different substrate bias voltages. (**a**) XRD patterns of WSe_2_ films prepared by selenization of W films with different substrate bias voltages. SEM images of WSe_2_ films prepared by selenization of W films with different substrate bias voltages (**b**) 0, (**c**) −100, (**d**) −150, and (**e**) −200 V.

The XRD patterns of the WSe_2_ films are shown in Fig. 3a. For all WSe_2_ films prepared under different conditions, the intensity of the (002) peaks is weaker than that of the (100) and (110) peaks.

The surface morphology of the WSe_2_ films prepared by selenization of the W films deposited at different substrate bias voltages is shown in Fig. 3b–e. When W film is deposited without a substrate bias voltage, the WSe_2_ presents a closely-packed lamellar surface and the nanosized platelets are embedded vertically in the matrix (Fig. 3b). As the substrate bias voltage increases from 0 to −100 V, the number of platelets embedded in the matrix decreases slightly. But the platelet size becomes larger (Fig. 3c). As the substrate bias voltage increases from −100 to −150 V, the number of platelets embedded in the matrix decreases significantly. Simultaneously, the WSe_2_ platelet size increases obviously and its size can reach the micron range (Fig. 3d). When the substrate bias voltage increases from −150 to −200 V, the number of small size platelet increases slightly (Fig. 3e).

### Effect of W film’s stress state on WSe_2_ film texture

The aforementioned experiments show that the number of platelets embedded in the matrix is affected by sputtering time, sputtering-gas pressure and substrate bias voltage. The high sputtering-gas pressure (2.0 Pa) is conducive to the growth of platelets embedded in the matrix. The effects of sputtering time are observed at a lower gas pressure (0.2 Pa). When the sputtering time is short (2 min), there are many small platelets on the surface of the WSe_2_ and most of the platelets are embedded vertically in the matrix. The long sputtering time (6 and 8 min) can inhibit vertical platelet growth, but there are many pores on the surface of WSe_2_ films and the WSe_2_ film peels off from the substrates. The impact of substrate bias voltage is studied by changing the bias under the same conditions at a higher gas pressure (0.9 Pa). The number of platelets decreases with increasing substrate bias voltage.

**Figure 4:**
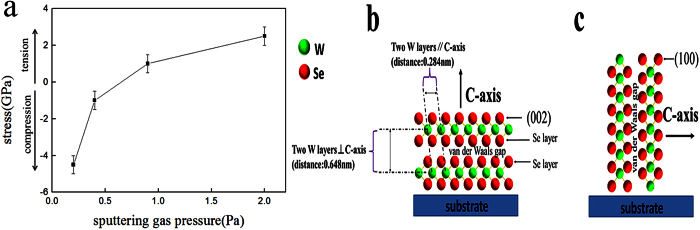
(**a**) Stress in W films as a function of the sputtering-gas pressure. WSe_2_ film texture: (**b**) WSe_2_ films with C-axis ⊥ substrate, (**c**) WSe_2_ films with C-axis // substrate.

Of the three factors (sputtering time, sputtering-gas pressure and substrate bias voltage), the sputtering-gas pressure is the most significant factor in our experiment. Residual stress of W film as a function of sputtering-gas pressure has been reported^16^,^17^. The magnetron sputtered W film undergoes a transition from compression to tension with increasing sputtering-gas pressure. In general, the stress state of the W film deposited at low pressure is a compressive stress. With increase in sputtering-gas pressure, the stress state of the film changes to a tensile stress. As the sputtering-gas pressure increases, the gas molecule density increases in a unit volume. The scattering phenomenon decreases the energy of the deposited particles as a result of collisions with the large number of gas molecules, which leads to a porous structure film. The compression decreases to form tension. The stress in the W films at different sputtering-gas pressures was measured and analyzed by the XRD sin^2^Ψ method^16^,^22^. Figure 4a shows the stress in W films as a function of sputtering-gas pressure. The W film deposited at 0.2 Pa exhibits a high compressive stress. As the sputtering-gas pressure is increased, the stress changes from compression to tension.

The residual stresses in W film with different thickness (20,40,60 and 80 nm) has been measured too. However, the diffraction peaks of this thin W film(<80 nm) is too weak for the XRD sin2Ψ method. So the W film thickness is too thin (<100 nm) for X ray residual stress measuring techniques. The stress variation law caused by thickness can’t be observed.

By combining the experimental phenomena with the stress states of the W film, it was found that a compressed W film deposited at low pressure is conducive to WSe_2_ film formation with few platelets embedded vertically in the matrix. A tensile W film that is deposited at a high pressure is conducive to WSe_2_ film formation with more vertical platelets. Therefore, this phenomenon depends on the stress states of the W film.

WSe_2_ films usually have two types of textures as shown in Fig. 4b,c 8,9. One is the C-axis ⊥ substrate texture with a (002) preferential orientation, whose van der Waals plane parallel to the substrate (Fig. 4b). The other is the C-axis // substrate, whose van der Waals plane perpendicular to the substrate (Fig. 4c). The orientation of the van der Waals gap determines the WSe_2_ film texture. When the van der Waals gap is parallel to the substrate, WSe_2_ film with C-axis ⊥ substrate can be prepared. So the (002) plane of the WSe_2_ crystal is exposed to the surface. When the van der Waals gap is perpendicular to the substrate, WSe_2_ film with C-axis // substrate can be prepared. The (100) plane of the WSe_2_ crystal is exposed to the surface. The platelets embedded in the matrix are WSe_2_ crystals with C-axis // substrate^7^.

The chemical reaction between the W film and Se vapor causes a serious volume expansion. Based on the WSe_2_ crystal structure with C-axis ⊥ substrate (Fig. 4b), the change from W to WSe_2_ crystal can be considered as the insertion of the Se atom layer and van der Waals gap. Two Se atom layers and a van der Waals gap are inserted between the two W atoms layer, which is perpendicular to the C-axis. The distance between the two layers of the W atoms, which are parallel to the van der Waals gap, is 0.648 nm (Fig. 4b). Only two Se atom layers are inserted between the two-layer W atoms along the C-axis. There is not van der Waals gap between them. The distance between the two layers of the W atoms, which is parallel to the C-axis, is only 0.284 nm. The Se atom layer and van der Waals gap between the two-layer W atoms cause the W film to expand from W to WSe_2_. Because 0.648 nm is much larger than 0.284 nm, the expanded direction is mainly along the C-axis of the WSe_2_ crystal. The expanded direction is also mainly along the C-axis of the WSe_2_ crystal for the other texture of the WSe_2_ with C-axis // substrate (Fig. 4c).

**Figure 5:**
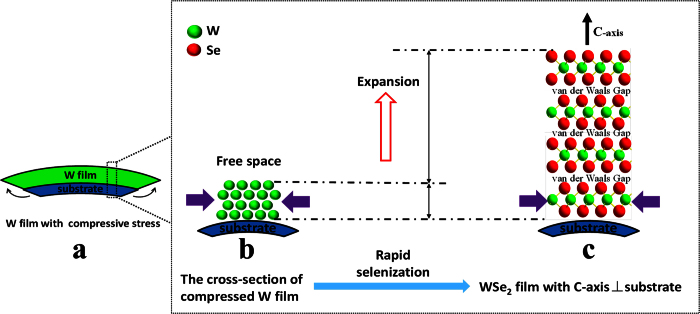
Schematic diagram of WSe_2_ film growth with C-axis ⊥ substrate by selenization of compressed W film. (**a**) W film with compressive stress, (**b**) cross-section of compressed W film, (**c**) WSe_2_ film with C-axis ⊥ substrate.

Figure 5a shows that the W film with compressive stress tends to expand along the film surface, where expansion is restrained by the substrate. Substrate deformation is caused by the expansion. The substrate exerts a force on the film, as shown by black arrows (Fig. 5a). The cross-section of the compressed W film is shown in Fig. 5b. One side of the W film is pushed by a force from the other side, which caused by the compressive stress. The compressive stress is shown by purple arrows (Fig. 5a). W atoms of the film are arranged closely under a compressive stress state. The compressive stress will block the horizontal expansion. The Se layer and van der Waals gap cannot be inserted in the vertical direction. However, the space above the film surface provides adequate free space for expansion, and the Se layer and van der Waals gap can be inserted horizontally into the W layers (Fig. 5b). The main expanded direction is shown by the red arrow (Fig. 5). Therefore the compressed W film is an important raw material of preparing WSe_2_ film with C-axis ⊥ substrate.

**Figure 6:**
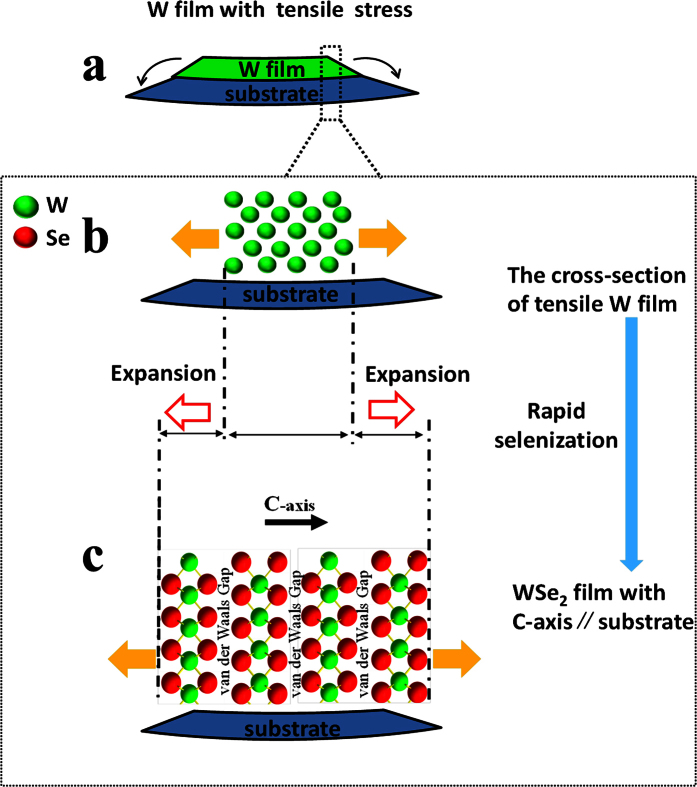
Schematic diagram of growth of WSe_2_ film with C-axis // substrate by selenization of tensile W film. (**a**) W film with tensile stress, (**b**) cross-section of tensile W film, (**c**) WSe_2_ film with C-axis // substrate.

As shown in Fig. 6a, the W film with tensile stress tends to shrink along the film surface. But the shrinkage will be restrained by the substrate. Substrate deformation is caused by shrinkage. The substrate exerts a force on the film, which is indicated by black arrows (Fig. 6a). The cross-section of the compressed W film is shown in Fig. 6b. One side of the W film is subjected to a stretching force from the other side based on the tensile stress. The tensile stress is shown by orange arrows (Fig. 6b). W atoms in the film are arranged loosely under a tensile stress state. The tensile stress and loose structure can help the insertion of a Se layer and a van der Waals gap, which is perpendicular to the substrate (Fig. 6b). The main expanded direction is shown by a red arrow (Fig. 6). Therefore the W film with tensile stress is an important raw material of preparing WSe_2_ film with C-axis // substrate.

## Discussion

The results have shown that the WSe_2_ texture depends on the W film precursor. The effect of sputtering time, sputtering gas-pressure, and substrate bias voltage on the texture of the WSe_2_ film was studied systematically. By combining the experimental phenomena with the stress states of the W film, it was shown that a compressed W film was deposited at low pressure, which is conducive to WSe_2_ film formation with few platelets embedded vertically in the matrix. Instead, a tensile W film deposited at a high pressure is conducive to WSe_2_ film formation with more platelets embedded vertically. The existence of compressive or tensile stresses in the W film is the main reason for WSe_2_ texture with C-axis ⊥ substrate or C-axis // substrate. Because platelets embedded in the matrix are WSe_2_ crystals with C-axis // substrate, a model for WSe_2_ film growth with different textures has been proposed. The inserting direction of the van der Waals gap is a key factor for the anisotropic formation of a WSe_2_ film: (i) for the W film with compressive stress, the van der Waals gap can be inserted horizontally into the W layers. Therefore the WSe_2_ film with C-axis ⊥ substrate existed mainly in the compressed W film. (ii) For the W film with tensile stress, the van der Waals gap can be inserted vertically into the W layer. Therefore the WSe_2_ film with C-axis // substrate existed mainly in the tensile W film. The findings herein will contribute to future fundamental research and engineering applications of other transition-metal dichalcogenide materials.

## Methods

W films were deposited on a quartz glass substrate (25 mm × 25 mm × 1 mm) with a low surface roughness (0.3 nm) by direct-current (DC) magnetron sputtering in Ar (99.999%). The substrates were cleaned using a standard procedure, which included 15 min each of ultrasonic cleaning in acetone and ethanol. The substrates were kept in ethanol and dried with nitrogen until they were loaded into the sputtering system. The deposition chamber was pumped down to a background pressure of 4.1 × 10^−4^ Pa. The W target purity was 99.999%. Substrates were mounted onto a rotating holder to achieve lateral film homogeneity. The distance between the target (Φ = 60 mm) and the substrate was 153 mm. The tungsten target was pre-sputtered for 5 min while the substrate was isolated from the plasma by using a shutter.

Following deposition, the W film was cut in half using a glass cutter. One half was used for detection and the other half was converted to WSe_2_ by exposing the W film to Se. All W films were heat-treated under the same selenization conditions. High-purity Se powder (0.7 g) was placed into a corundum crucible (3 ml). Figure 1 shows the experimental setup for the selenization process. The precursor W film and corundum crucible were placed in a sliding furnace. Both sides of the corundum crucible were sealed with a quartz plug. The furnace was pumped down to a background pressure of 1.0 Pa before it was filled with protective vapor (N_2_ 99.999%). This process was repeated three times to remove residual oxygen in the vacuum system. Finally, N_2_ was added into the furnace until the vapor pressure reached 0.04 MPa. After the furnace temperature had reached 600 °C, it was pushed to the other side of the flat slideway to encounter the sample. The sample was maintained at this temperature for 30 min. The furnace was cooled to 500 °C at 10 °C/min. Finally, the furnace was turned off and the samples were cooled naturally.

X-ray diffractometry (XRD) was used to characterize the orientation and phase microstructure (Rigaku Ltd., Japan, D/max 2550VB, CuK radiation, U = 40 kV, I = 250 mA). Field-emission scanning electron microscopy (NOVA nano SEM 230) was used to study the W and WSe_2_ film morphology. Thin films for the high-resolution transmission electron microscopy (HRTEM, JEOL 2010II) study were obtained by peeling off films from the quartz substrate by immersing them in dilute HF (~1%). Because of the weak adhesion to the substrate, the films peeled easily from the substrate, and were transferred to a standard (amorphous carbon-covered) copper grid for HRTEM analysis. The residual stress of the film was measured by using the XRD sin^2^Ψ method (Bruker D8 Discover).

## Additional Information

**How to cite this article**: Li, H. *et al*. Effect of magnetron sputtering parameters and stress state of W film precursors on WSe_2_ layer texture by rapid selenization. *Sci. Rep.* **6**, 36451; doi: 10.1038/srep36451 (2016).

**Publisher’s note:** Springer Nature remains neutral with regard to jurisdictional claims in published maps and institutional affiliations.

## Figures and Tables

**Table 1:** Conditions used for deposition of W films by DC magnetron sputtering.

Sample No.	Sputtering power (W)	P_Ar_ (Pa)	Sputtering time (min)	Thickness of W film(nm)
1	120	0.2	**2**	20
2	120	0.2	**4**	40
3	120	0.2	**6**	60
4	120	0.2	**8**	80

**Table 2:** Conditions used for deposition of W films by DC magnetron sputtering.

Sample No.	Sputtering power (W)	P_Ar_ (Pa)	Sputtering time (min)
1	120	2.0	10
	120	**0.2**	25
2	120	2.0	10
	120	**0.4**	20
3	120	2.0	10
	120	**0.9**	11
4	120	2.0	10
	120	**2.0**	10

**Table 3:** Conditions used for W film deposition by DC magnetron sputtering.

Sample No.	Sputtering power (W)	P_Ar_ (Pa)	Sputtering time (min)	Substrate bias voltage(V)
1	120	2.0	10	
	120	0.9	10	**0**
2	120	2.0	10	
	120	0.9	10	**−100**
3	120	2.0	10	
	120	0.9	10	**−150**
4	120	2.0	10	
	120	0.9	10	**−200**
